# Modulation of programmed cell death by botanical drugs in Alzheimer’s disease: a review from a traditional Chinese medicine perspective

**DOI:** 10.3389/fphar.2026.1811185

**Published:** 2026-04-22

**Authors:** Boyu Li, Ziheng Cui, Yanji Xu, Meihua Xu

**Affiliations:** Medical College of Yanbian University, Yanji, Jilin, China

**Keywords:** Alzheimer′s disease, apoptosis, autophagy, ferroptosis, programmed cell death, pyroptosis, traditional Chinese medicine

## Abstract

Alzheimer’s disease (AD) involves dysregulation of programmed cell death (PCD) pathways, such as apoptosis, ferroptosis, autophagy, and pyroptosis. Current therapeutic options are limited, prompting interest in multi-target regulators such as metabolites derived from traditional Chinese medicine (TCM) botanical drugs. This systematic review critically evaluates recent studies on TCM-derived metabolites that modulate PCD in AD models. We identify key limitations: many metabolites are pan-assay interference metabolites (PAINS) with questionable pharmacological relevance; preclinical models inadequately recapitulate sporadic AD; and translational challenges persist in bioavailability and brain targeting. Future research requires orthogonal validation, improved delivery systems, and stage-specific strategies. This review provides a critical foundation for the development of TCM-inspired therapies for AD.

## 1 Introduction

Alzheimer’s disease (AD), also known as senile dementia, is the most common neurodegenerative disease and a leading cause of dementia. Its typical features include insidious onset with progressive memory decline as the core symptom and slowly progressive cognitive dysfunction (Breijyeh and Karaman, 2020). The pathogenesis of AD is complex, involving abnormal deposition of β-amyloid protein (Aβ), hyperphosphorylation of tau protein, and others (Zheng and Wang, 2025). Currently, mainstream therapies can only offer limited symptom relief and are difficult to effectively block or reverse disease progression, facing both efficacy controversies and safety challenges. Therefore, exploring new therapeutic strategies is urgent. Recent studies have found that multiple programmed cell death (PCD) pathways play a key role in the occurrence and development of AD (Fink et al., 2020). In this context, natural products, which have historically been a rich source of bioactive metabolites, offer a vast reservoir for drug discovery. Traditional Chinese medicine (TCM), as one of the most systematically documented and continuously practiced medical systems in the world, provides a unique theoretical framework and extensive empirical knowledge for the application of these natural products. Many bioactive metabolites, such as alkaloids, glycosides, and terpenoids, are not exclusive to TCM but are widely distributed in medicinal plants across different cultures. However, the value of TCM in modern AD research lies in its holistic therapeutic principles and centuries of clinical experience, which offer a powerful lens for identifying and validating multi-target interventions from nature. This article reviews the recent progress in research on natural products, guided by TCM principles, in intervening in AD through the regulation of apoptosis, ferroptosis, autophagy, and pyroptosis, aiming to provide theoretical foundations and practical insights for exploring new TCM-inspired strategies for preventing and treating AD.

## 2 Literature retrieval and screening methods

### 2.1 Search strategy

A systematic literature search was performed in PubMed and Web of Science, covering publications up to January 2026. A combination of Medical Subject Headings (MeSH) terms and keywords was employed. The core search strategy for PubMed was as follows:

#1 Alzheimer’s disease (“Alzheimer disease” [Mesh] OR “Alzheimer*” [Title/Abstract] OR “AD” [Title/Abstract])

#2 Programmed cell death (“Apoptosis” [Mesh] OR “apoptosis” [Title/Abstract] OR “Ferroptosis” [Mesh] OR “ferroptosis” [Title/Abstract] OR “Autophagy” [Mesh] OR “autophagy” [Title/Abstract] OR “Pyroptosis” [Mesh] OR “pyroptosis” [Title/Abstract] OR “programmed cell death” [Title/Abstract])

#3 Traditional Chinese medicine (“Medicine, Chinese Traditional” [Mesh] OR “traditional Chinese medicine” [Title/Abstract] OR “TCM” [Title/Abstract] OR “herbal medicine” [Title/Abstract] OR “Chinese botanical drug” [Title/Abstract] OR “botanical drug” [Title/Abstract] OR “plant extract” [Title/Abstract] OR “natural product” [Title/Abstract] OR “phytochemical” [Title/Abstract])

#4 Combination: #1 AND #2 AND #3.

### 2.2 Inclusion and exclusion criteria

Studies were included if they met the following criteria: (1) original research investigating TCM-derived natural products in AD via PCD pathways; (2) peer-reviewed original articles; (3) published in English or Chinese.

Studies were excluded if they met any of the following criteria: (1) duplicate publications; (2) irrelevant to the topic after full-text assessment; (3) publications with limited contemporary relevance due to extended time since publication.

### 2.3 Study selection and data extraction

The literature search and screening were conducted by the first author. Titles and abstracts of all retrieved records were screened to identify potentially eligible studies. Full texts of these studies were then assessed for final inclusion according to the predefined criteria. Data from the included studies were extracted by the first author using a standardized form.

## 3 Apoptosis and AD

**FIGURE 1:**
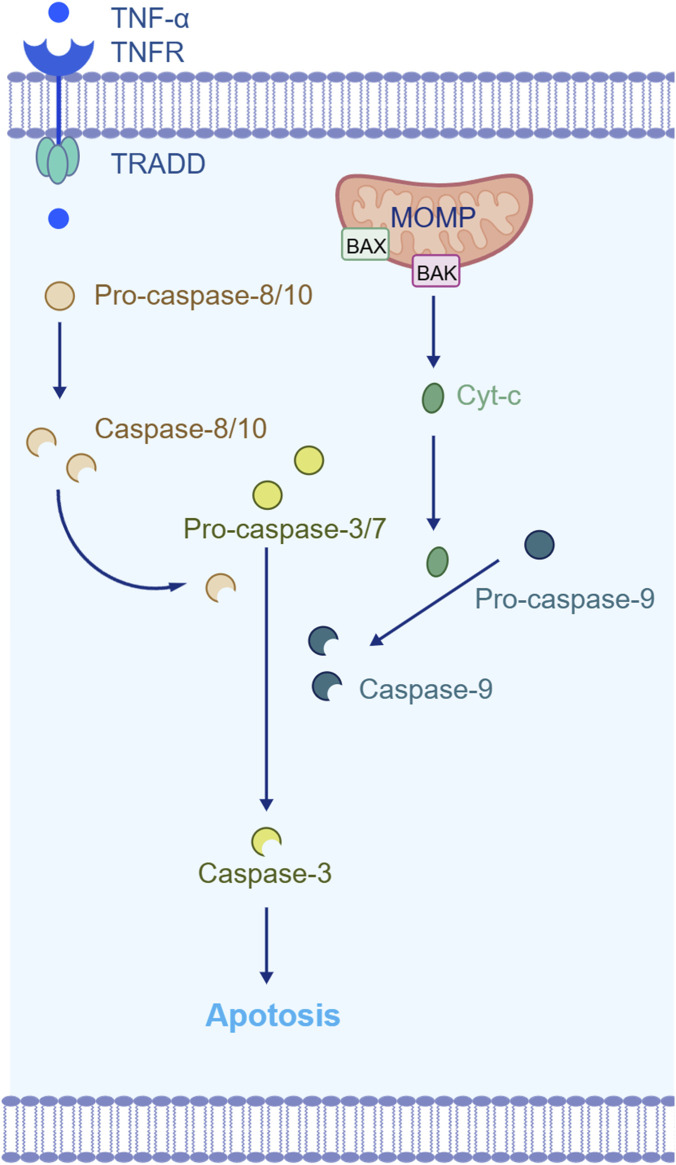
Mechanisms of apoptosis. Created with BIOGDP.com (Jiang et al., 2025), licensed under CC BY NC.

Under physiological conditions, apoptosis is a key mechanism for maintaining tissue homeostasis, with the intrinsic pathway initiated by mitochondria and the extrinsic pathway mediated by death receptors (Newton et al., 2024) (Figure 1). Under various stress or inflammatory conditions, Bcl-2-associated X protein (BAX) translocates to mitochondria, altering mitochondrial membrane permeability and releasing pro-apoptotic factors such as cytochrome C (Cyt-C) into the cytoplasm, thereby activating caspase-9 and its downstream effectors caspase-3/7 to induce intrinsic apoptosis. The extrinsic pathway is triggered by the binding of death ligands to receptors, which forms a death-inducing signaling complex that activates the caspase cascade (Bertheloot et al., 2021). In AD, Aβ can disrupt neuronal homeostasis by regulating the balance between pro-apoptotic and anti-apoptotic factors, and the degree of neuronal apoptosis correlates positively with the severity of cognitive impairment (Sharma et al., 2021; Rajesh and Kanneganti, 2022). Therefore, intervening in apoptosis is an important direction in AD treatment research (Table 1).

**TABLE 1:** Regulation of neuronal apoptosis by botanical drugs.

Botanical drug/Metabolite	Model system	Dose/Concentration	Treatment duration	Mechanism and outcome	Controls used	References
Berberine	*In vitro*, Aβ25-35-treated BV2, N2a cells	5 μM (protective); 10, 20 μM (toxic	24 h co-treatment	Regulated miR-188/NOS1 axis → ↑Cell viability, ↓apoptosis	Vehicle, transfection controls	Chen et al. (2020a)
Berberine	*In vitro*, Aβ25-35-treated HPN, SK-N-SH cells	1, 10 μM	2 h pre +24 h co-treatment	Regulated BACE1-AS/miR-132-3p axis → ↑Cell viability, ↓LDH, ROS, apoptosis	Vehicle, transfection controls	Ge et al. (2020)
Berberine	*In vitro*, Aβ42-treated HN cells	0.5, 1, 2 μM (protective); 20 μM (toxic)	24 h co-treatment	Regulated circHDAC9/miR-142-5p axis → ↓apoptosis, ↓inflammatory cytokines	Untreated control, vehicle, transfection controls	Zhang et al. (2020)
Ginsenoside Ro	*In vivo*, APP/PS1 mice	5, 15 mg/kg (oral)	1 month	Regulated IBA1/GFAP-MAPK → ↓Aβ, ↓neuroinflammation, ↓cytokines, ↑IL-10, ↓apoptotic proteins, ↑cognition, ↑neuronal survival	WT, model, ginseng extract	Li et al. (2025)
Ginsenoside CK	*In vitro*, Aβ42-HT22 cells; *In vivo*, scopolamine mice	*In vitro*: 2.5–10 μM; *In vivo*: 20, 40 mg/kg	*In vitro* 24 h; *In vivo* 14 d	Regulated Nrf2/Keap1, bound Aβ42, modulated APP enzymes → ↓Aβ, ↓ROS, ↓apoptotic proteins, ↑cognition	Vehicle, model, memantine	Li et al. (2023a)
Ginsenoside Rg1	*In vivo*, tree shrew AD model	30 mg/kg (oral)	6 weeks	↓Bax, ↑Bcl-2, ↓BACE1, ↓Aβ1-42, ↓p-tau, ↑MAP2/NeuN, modulated gut microbiota, ↑cognition	Model, blank	Guo et al. (2021)
Ginsenoside Rg2	*In vivo*, Aβ25-35 intrahippocampal injection rats	25, 50, 100 mg/kg (oral)	6 weeks	Activated PI3K/Akt, ↑Bcl-2/Bax, ↓caspase-3, ↓neuronal apoptosis, ↑cognitive function	Model group, positive control donepezil	Cui et al. (2021)
Artemisinin	*In vitro*: Aβ1-42-induced BV2 cells; *In vivo*: Aβ1-42 hippocampal injection C57 mice	*In vitro*: 20 μM; *In vivo*: 5 mg/kg (i.p.)	*In vitro*: 2 h + 24 h; *In vivo*: 4 weeks pre-modeling	Inhibited TLR4/NF-κB, ↓pro-inflammatory cytokines, ↓ROS/iNOS/COX2, ↓microglial migration, ↓astrocyte activation, ↓neuronal apoptosis, ↑cognitive function	Negative controls: untreated, vehicle, model	Zhao et al. (2022)
Tanshinone IIA	*In vivo*: APP/PS1 mice	15, 30 mg/kg (i.p.)	4 weeks	Activated PI3K/Akt, inhibited GSK-3β, ↓tau phosphorylation, ↓neuronal apoptosis, ↓AChE, ↑ChAT, ↓oxidative stress, ↑cognitive function	Model group, WT control	Peng et al. (2022a)
Icariin + Tanshinone IIA	*In vivo*: APP/PS1 mice	10 mg/kg (i.p.)	40 days	Brain-targeted delivery, ↓Aβ deposition, ↓neuroinflammation, ↓oxidative stress, ↓apoptosis, ↑synaptic protein SYN, ↑cognitive function	Model group, free drug, unmodified liposomes	Wang J et al. (2023)
Shenghui Decoction	*In vitro*: Aβ1-42-induced N2a cells; *In vivo*: APP/PS1 mice; *C. elegans*	*In vitro*: 25–200 μg/mL; *In vivo*: 6.75, 13.5, 27 mg/kg (oral); *C. elegans*: 200–800 μg/mL	*In vitro* 24 h; *In vivo* 2 months	Inhibited PDE4B, ↑cAMP/PKA/CREB, ↑Bcl-2, ↓Bax/cleaved caspase-3, ↓Aβ deposition, ↑cognitive function	Model group, positive control donepezil	Gao et al. (2025)
Total flavonoids, derived from Cynomorii Herba (Cynomorium songaricum Rupr., Cynomoriaceae)	*In vivo*, Aβ1-42 hippocampal injection rats	25, 50, 100 mg/kg (oral)	28 days	Activated BDNF/TrkB, ↑Bcl-2, ↓Bax/caspase-3/caspase-9, ↑ChAT/ACH, ↓AChE, ↓Aβ deposition, ↑cognitive function	Model group, positive control donepezil	Gu et al. (2023)
Quercetin, a flavonol metabolite distributed in multiple botanical families (e.g., Sophora japonica L., Fabaceae)	*In vivo*, APP/PS1 mice	100 mg/kg (oral)	6 months	Activated Keap1/Nrf2/HO-1, ↑SOD/CAT/GSH, ↓MDA/ROS, ↓Bax/caspase-3, ↑Bcl-2, ↑ACh, ↓AChE, ↓Aβ deposition, ↑cognitive function	Model group, positive control donepezil	Cheng et al. (2024a)
Ginsenoside Rg1	*In vivo*, tree shrew AD model	7.5, 15, 30 mg/kg (oral)	8 weeks	Regulated Wnt/GSK-3β/β-catenin, ↓BACE1, ↓Aβ1-42, ↓p-Tau, ↑antioxidant enzymes, ↓MDA, ↓inflammation, ↑Bcl-2/Bax, ↓caspase-3, ↑MAP2/NeuN, ↑cognitive function	Model group, positive control donepezil	Yang et al. (2022b)
Notoginsenoside R2, derived from Notoginseng Radix et Rhizoma (Panax notoginseng (Burk.) F.H.Chen, Araliaceae)	*In vitro*: primary rat cortical neurons; *In vivo*: SAMP8 mice, Aβ25-35-induced rats	*In vitro*: 30 μM; *In vivo*: 250 mg/kg (i.p.)	*In vivo* 20 weeks	Regulated miR-27a/SOX8/β-catenin, ↓apoptosis, ↓inflammation (↓COX-2), ↑cognitive function	Model group, miR-27a antagomir	Hu et al. (2021)
Tanshinone IIA	*In vivo*, APP/PS1 mice	15, 30 mg/kg (i.p.)	4 weeks	Regulate the PI3K/Akt/GSK-3β axis, ↓ tau protein phosphorylation, ↑cognitive function	Model group, WT control	Peng et al. (2022b)
Sennoside A, derived from Rhei Radix et Rhizoma (Rheum palmatum L., Polygonaceae)	*In vivo*: APP/PS1 mice; *In vitro*: LPS-induced BV2 cells	*In vivo*: 7.5, 15, 30 mg/kg (drinking water); *In vitro*: 12.5–800 μM	*In vivo* 8 weeks; *In vitro* 24 h	Inhibited TRAF6/NF-κB, ↓apoptosis, ↓ferroptosis, ↓oxidative stress, ↓inflammatory cytokines, ↑cognitive function	Model group, positive control donepezil, TRAF6 overexpression/knockdown	Li et al. (2023c)
Schisandrin A (Schisandrae Chinensis Fructus, Schisandra chinensis (Turcz.) Baill., Schisandraceae)	*In vitro*, Aβ25-35-induced SH-SY5Y, SK-N-SH cells	1, 5, 10, 15 μg/mL	24 h	Activate the ERK/MAPK pathway, ↑p-ERK1/2	Model group, ERK activator LM22B-10	Jia et al. (2024)

### 3.1 Effects of botanical drugs derived from TCM on AD via apoptosis

#### 3.1.1 Alkaloids

Berberine (BBR) is a natural alkaloid metabolite known for its anti-inflammatory, antioxidant, and neuroprotective effects.It is predominantly derived from Coptidis Rhizoma (*Coptis chinensis* Franch., Ranunculaceae) and is also found in other plant species worldwide, known for its anti-inflammatory, antioxidant, and neuroprotective effects. Studies have found that BBR can intervene in core pathological aspects of AD by regulating microRNAs (miRNAs). In Aβ-related models, BBR inhibits caspase-3 activity and exerts anti-apoptotic effects by activating the miR-188/neuron-specific nitric oxide synthase (NOS1) pathway or regulating miR-132-3p and miR-142-5p (Chen M. et al., 2020; Ge et al., 2020; Zhang et al., 2020).

#### 3.1.2 Glycosides

Various ginsenosides, a class of glycoside metabolites derived from *Panax ginseng C.A.Mey.*, exert neuroprotective effects through different mechanisms. Ginsenoside Ro, a glycoside metabolite derived from Ginseng Radix et Rhizoma (*P. ginseng* C.A.Mey., Araliaceae), alleviates Aβ deposition and significantly inhibits the abnormal activation of ionized calcium-binding adaptor molecule 1 (Iba1)-positive microglia and glial fibrillary acidic protein (GFAP)-positive astrocytes, thereby improving the neuroinflammatory environment, with a decrease in pro-inflammatory factor levels and an increase in anti-inflammatory IL-10 expression. Simultaneously, it effectively inhibits neuronal apoptosis by regulating Bax/Bcl-2/Caspase-3 expression, and its mechanism of action is related to the phosphorylation levels of key molecules such as p38/JNK, reflecting its synergistic neuroprotective role through modulation of the neuroinflammation-cell death interaction network (Li et al., 2025). Ginsenoside Compound K (CK), a glycoside metabolite derived from Ginseng Radix et Rhizoma (*P. ginseng* C.A.Mey., Araliaceae), regulates Aβ metabolism, activates the Nrf2/Keap1 pathway to inhibit oxidative stress, and improves synaptic function by modulating the Cyt-C/caspase-3 apoptosis axis (Li N. et al., 2023). Ginsenoside Rg1, also derived from Ginseng Radix et Rhizoma (*P. ginseng* C.A.Mey., Araliaceae), exerts broad neuroprotective effects. It not only reduces Aβ deposition and tau protein phosphorylation, modulates apoptosis-related proteins to alleviate neuronal damage, but also reshapes the gut microbiota to regulate energy metabolism and neuroinflammation through the gut-brain axis (Guo et al., 2021). Ginsenoside Rg2, a glycoside metabolite derived from Ginseng Radix et Rhizoma (*P. ginseng* C.A.Mey., Araliaceae), antagonizes neuronal apoptosis by activating the PI3K/Akt signaling pathway, upregulating Bcl-2, downregulating Bax, and inhibiting caspase-3 activity (Cui et al., 2021).

#### 3.1.3 Terpenoids

Artemisinin, a terpenoid metabolite derived from Artemisiae Annuae Herba (*Artemisia annua* L., Asteraceae),is isolated from Artemisia annua by Chinese scholars, with effects in the prevention and treatment of malaria, AD, and other conditions. In Aβ-stimulated cells, artemisinin reduces reactive oxygen species (ROS), inducible nitric oxide synthase, and inflammatory factors, and decreases neuronal apoptosis and microglial activation by blocking the Toll-like receptor 4/nuclear factor κB (TLR4/NF-κB) pathway, thereby improving cognitive impairment in AD mice (Zhao et al., 2022). Studies have shown that Tanshinone IIA, a terpenoid metabolite derived from Salviae Miltiorrhizae Radix et Rhizoma (*Salvia miltiorrhiza* Bge., Lamiaceae),upregulates the expression of phosphorylated phosphoinositide 3-kinase (p-PI3K) and protein kinase B (p-Akt), promotes phosphorylation of glycogen synthase kinase-3β (GSK-3β), inhibits its activity, and reduces tau protein hyperphosphorylation, thereby reducing hippocampal neuronal apoptosis (Peng et al., 2022a). Notably, Angiopep-2-modified Icariin (a glycoside metabolite derived from Epimedii Folium, *Epimedium brevicornu* Maxim., Berberidaceae) and tanshinone IIA co-delivered liposomes efficiently cross the blood-brain barrier and target brain tissue, significantly improving AD-related pathological changes by co-regulating the Bax/Bcl-2/caspase-3 signaling pathway (Wang Q et al., 2023). Although this brain-targeted strategy offers a promising approach for delivering bioactive TCM metabolites, the underlying synergistic mechanisms require *in vitro* validation, and clinical translation faces challenges in scalable manufacturing and oral bioavailability. Future studies should integrate pharmacokinetic and multi-omic analyses to elucidate targeting mechanisms and optimize administration routes.

### 3.2 The effect of traditional Chinese medicine formulas on AD via apoptosis

Shenghui Decoction, derived from Bianzheng Lu, contains active metabolites that inhibit phosphodiesterase 4B (PDE4B) activity, reduce the hydrolysis of cyclic adenosine monophosphate (cAMP), activate protein kinase A (PKA), and promote the phosphorylation of cAMP response element-binding protein, thus upregulating the expression of Bcl-2, brain-derived neurotrophic factor (BDNF), and nerve growth factor (NGF), thereby inhibiting neuronal apoptosis (Gao et al., 2025). Serum pharmacological studies indicate that Danggui Shaoyao San (DSS) inhibits tau protein hyperphosphorylation and activates the cAMP/PKA/CREB signaling axis, alleviating mitochondrial calcium overload in hippocampal neurons, inhibiting Cyt-c release, downregulating the BAX/Bcl-2 ratio, and caspase-3 activation, ultimately blocking neuronal programmed cell death (PCD) pathways (Zhang et al., 2025). These formulas all demonstrate the overall intervention advantage of multi-target anti-apoptosis. It is worth noting that various formulas such as Shenqi Pills, Qingxin Kaizao Decoction, Dabuyuan Decoction, and Dioscorea Pills share a common mechanism of inhibiting apoptosis by activating the PI3K/AKT pathway (Liu S. et al., 2024; Ma et al., 2025).

In summary, TCM intervenes in AD-related apoptosis through a multi-layered mechanistic network. At the upstream signaling level, TCM activates survival pathways including PI3K/Akt and cAMP/PKA/CREB to enhance neuronal viability. At the cellular stress level, TCM suppresses oxidative stress and neuroinflammation via Nrf2 and TLR4/NF-κB pathways, thereby eliminating apoptotic triggers. At the core execution level, TCM directly regulates the Bcl-2/BAX/caspase-3 axis to block the final execution of apoptosis. Additionally, certain alkaloids exert anti-apoptotic effects through miRNA regulation, while various terpenoids primarily target inflammatory pathways, demonstrating the mechanistic diversity of TCM metabolite. Among these, the PI3K/Akt pathway emerges as a convergent node for multiple TCM formulas, highlighting its centrality in TCM-mediated neuroprotection against apoptosis.

## 4 Ferroptosis and AD

**FIGURE 2:**
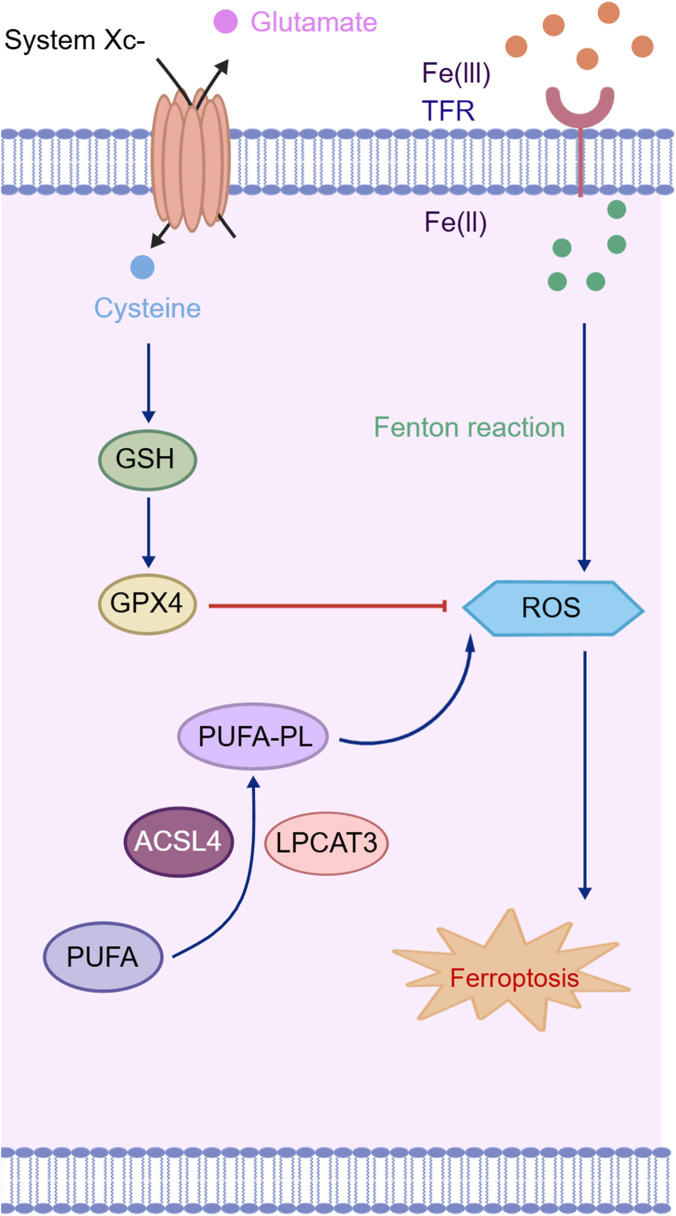
Mechanisms of ferroptosis. Created with BIOGDP.com (Jiang et al., 2025), licensed under CC BY NC.

Ferroptosis is a novel form of programmed cell death (PCD) characterized by iron accumulation and lipid peroxidation. Its execution is governed by the dysregulation of three interconnected axes: iron metabolism, the antioxidant defense system, and lipid peroxidation (Li J. et al., 2020) (Figure 2). Studies have confirmed that there is significant abnormal iron accumulation in the brains of AD patients, particularly in the hippocampus. This phenomenon is closely related to the disruption of brain iron homeostasis: the iron uptake mediated by Transferrin Receptor one on the surface of neurons is continuously enhanced, while the function of membrane iron transporters responsible for iron efflux is relatively insufficient. This dual imbalance between uptake and efflux leads to an abnormal expansion of the intracellular free iron pool, forming a toxic foundation for subsequent damage. Abnormal iron accumulation catalyzed by the Fenton reaction generates large amounts of ROS, promoting lipid peroxidation and initiating ferroptosis (Chavoshinezhad et al., 2023). Secondly, dysfunction of the antioxidant defense system, particularly the glutathione (GSH)-glutathione peroxidase 4 (GPX4) axis, is a key element. GPX4 is responsible for reducing phospholipid hydroperoxides (PL-OOH) to harmless alcohols. Its loss of activity leads to uncontrolled lipid peroxidation, triggering ferroptosis (Liu and Chen, 2024). Ultimately, under further catalysis by lipoxygenases (LOXs), polyunsaturated fatty acids undergo lipid peroxidation, generating large amounts of toxic products. When the defense system, including GPX4, fails, these lipid peroxides accumulate and damage the integrity of the cell membrane, ultimately destroying the membrane integrity and directly inducing neuronal death (Hambright et al., 2017; Wang J et al., 2023). Research has shown that inhibition of GPX4 function in AD exacerbates lipid peroxidation and induces neuronal ferroptosis, while overexpression of GPX4 or the use of ferroptosis inhibitors can alleviate Aβ toxicity (Dar et al., 2024). In conclusion, regulating ferroptosis plays an important role in AD treatment (Table 2).

**TABLE 2:** Regulation of ferroptosis by botanical drugs.

Botanical drug/Metabolite	Model system	Dose/Concentration	Treatment duration	Mechanism and outcome	Controls used	References
Berberine	*In vivo*, 3×Tg-AD mice; *In vitro*, RSL3-induced N2a-sw cells	*In vivo*: 50 mg/kg (oral); *In vitro*: 1–15 μM	*In vivo* 7.5 months; *In vitro* 24 h	Bound Nrf2/Keap1, activated AMPK/GSK3β/Nrf2, ↑SLC7A11/GPX4/FPN1, ↓TFR/FTH1, ↓iron levels, ↓lipid peroxidation, ↓Aβ/tau pathology, ↑cognitive function	Model group, positive control Fer-1, Nrf2 inhibitor ML385	Li et al. (2023b)
Salidroside	*In vivo*, SAMP8 mice	50, 100 mg/kg (oral)	12 weeks	Activated Nrf2/GPX4, ↓iron deposition, ↓oxidative stress, ↓CD8+ T cell infiltration, ↓inflammatory cytokines, ↑IL-10, ↓Aβ deposition, ↑cognitive function	Model group, positive control Fer-1	Yang et al. (2023)
Salidroside	*In vitro*: Glu-induced HT22 cells; *In vivo*: Aβ1-42-induced WT and Nrf2^−/−^ mice	*In vitro*: 40 μM; *In vivo*: 50 mg/kg (oral)	*In vitro* 24 h; *In vivo* 75 days	Activated Nrf2/HO1, ↑GPX4/SLC7A11/NQO1, ↓PTGS2, ↓Fe^2+^/ROS/lipid peroxidation, ↑MMP, ↑cognitive function	Model group, positive control Fer-1, siRNA Nrf2, Nrf2^−/−^ mice	Yang et al. (2022a)
Forsythoside A	*In vitro*: Aβ1-42-induced N2a cells, erastin-induced HT22 cells, LPS-induced BV2 cells; *In vivo*: APP/PS1 mice	*In vitro*: 40, 80 μM; *In vivo*: 30 mg/kg (oral)	*In vitro* 24 h; *In vivo* 42 days	Activated Nrf2/GPX4, ↓iron deposition, ↓lipid peroxidation, ↓inflammatory cytokines, ↑cognitive function	Model group, positive control Fer-1, siRNA GPX4/Nrf2	Wang et al. (2022a)
Senegenin	*In vitro*, Aβ25-35-induced PC12 cells	60 μM	24 h	↓ACSL4/PEBP1, ↑GPX4, ↓ROS/MDA, ↑GPX activity, improved mitochondrial function	Model group, positive control Fer-1	Zhang et al. (2022)
Curculigoside	*In vivo*: scopolamine, OA-induced mice; *In vitro*: SH-SY5Y cells	*In vivo*: 12.5,25,50 mg/kg (scopolamine); 25,50,100 mg/kg (OA); *In vitro*: 3.125 μM (SLC7A11),12.5 μM (GPX4)	*In vivo* 7d+4d (scopolamine); 7d + 22d+8d (OA); *In vitro* 24 h	Regulated SLC7A11/GPX4 pathway, ↑GSH/GSSG/GPX4/GCLM/GCLC/Fpn, ↓SLC7A11/ACSL4/STEAP3/DMT1, ↓iron/MDA/ROS, ↓Aβ/p-tau, ↑cognitive function	Model group, Erastin (SLC7A11 inhibitor), RSL3 (GPX4 inhibitor), Se (GPX4 agonist)	Gong et al. (2024)
Paeoniflorin	*In vivo*: APP/PS1 mice; *In vitro*: Erastin-induced PC12 cells	*In vivo*: 5, 10 mg/kg (oral); *In vitro*: 2.5, 5 μM	*In vivo* 5 weeks; *In vitro* 24 h	Bound p53, regulated p53/SLC7A11/GPX4 axis, ↓iron/MDA, ↑SOD, ↑JC-1, ↓neuronal damage, ↑cognitive function	Model group, p53 ASO	Zhai et al. (2023)
Astragalus saponins	*In vivo*: SAMP8 mice; *In vitro*: Erastin-induced HT22 cells	*In vivo*: 200,400,800 mg/kg (oral); *In vitro*: 50,100 μg/mL	*In vivo* 12 weeks; *In vitro* 24 h	Inhibited NOX4, activated Nrf2/HO-1, ↑SLC7A11/GPX4/FTH1/FPN1, ↓Fe^2+^/MDA/ROS, ↑GSH/SOD, ↓Aβ/p-Tau, ↑cognitive function	Model group (SAMR1), positive control donepezil, Fer-1, NOX4 overexpression	Wang et al. (2025a)
Artemisinin	*In vitro*: HT22, SH-SY5Y, primary neurons; *In vivo*: IKE hippocampal injection mice	*In vitro*: 5–20 μM; *In vivo*: 5,10 mg/kg (i.p.)	*In vitro* 24 h; *In vivo* 5 d	Targeted KEAP1, activated Nrf2-SLC7A11-GPX4, ↓lipid peroxidation, ↑GSH, improved mitochondrial morphology, ↑cognitive function	Model group, Fer-1, siNrf2, sgSLC7A11/GPX4, KEAP1 overexpression	Deng et al. (2025)
Danggui Shaoyao San (formula)	*In vivo*, APP/PS1 mice	7.5, 15, 30 g/kg (oral)	30 d	Activated AMPK, inhibited Sp1/ACSL4, ↑FTH/GPX4, ↓Fe^2+^/MDA/4-HNE, ↑cognitive function	Model group, positive control donepezil	Gong et al. (2025)
Linggui Zhugan Decoction (formula)	*In vivo*, APP/PS1 mice	*In vivo*, APP/PS1 mice	4 weeks	Regulated AMPK/p53/SLC7A11/GPX4, ↓Fe^2+^/MDA, ↑GSH, ↑NeuN^+^/GPX4^+^ cells, improved mitochondrial morphology, ↓iron deposition, ↑cognitive function	Model group, positive control donepezil	Fan et al. (2024)
Ginkgolide B (from Ginkgo biloba L., Ginkgoaceae)	*In vitro*, Aβ1-42-induced N2a cells	100 μM	2 h pre +24 h	Regulated SPP1/FTH1, ↓SPP1, ↑FTH1, ↓apoptosis, ↓oxidative stress, ↓Aβ levels	Model group	Zhang et al. (2023b)
Sennoside A (from Rheum palmatum L., Polygonaceae)	*In vivo*: APP/PS1 mice; *In vitro*: LPS-induced BV2 cells	*In vivo*: 7.5,15,30 mg/kg (drinking water); *In vitro*: 12.5–800 μM	*In vivo* 8 weeks; *In vitro* 24 h	Inhibited TRAF6/NF-κB, ↓apoptosis, ↓ferroptosis, ↓oxidative stress, ↓inflammatory cytokines, ↑cognitive function	Model group, positive control donepezil, TRAF6 overexpression/knockdown	Li et al. (2023d)
Chrysophanol (from Rheum palmatum L., Polygonaceae)	*In vivo*: Aβ25-35+D-galactose-induced rats; *In vitro*: Aβ25-35-induced PC12 cells	*In vivo*: 0.036 mg/mL (oral); *In vitro*: 25,50 μM	*In vivo* 28 d; *In vitro* 12 h pre +24 h	↑GPX4/GPx activity/GSH, ↓ROS/LPO/MDA, ↓ferroptosis, improved mitochondrial morphology, ↑cognitive function	Model group, positive control donepezil, Fer-1	Luo et al. (2023)
Betaine (multiple plants)	*Ex vivo*, Aβ1-42-treated rat brain synaptosomes	*In vivo*: 250 mg/kg (oral)	*In vivo* 21 d	↓TfR1/ACSL4/MDA/8-OHdG/total iron, ↑GPX4/GSH, regulated iron metabolism, inhibited ferroptosis	Model group, boric acid group	Hacioglu et al. (2024)
Schisandra lignans (from Schisandra chinensis (Turcz.) Baill., Schisandraceae)	*In vivo*: SAMP8 mice; *In vitro*: Erastin-induced HT22 cells	*In vivo*: 100,200 mg/kg (oral); *In vitro*: 7.5,30 μg/mL	*In vivo* 10 weeks; *In vitro* 24 h	Activated Nrf2/FPN1 pathway, ↑GPX4/SLC7A11, ↓FACL4, ↓TFR/DMT1, ↑FTH1, ↓Fe^2+^/ROS, ↑MMP, ↓p-Tau/APOE/APP, ↑cognitive function	Model group, positive control Fer-1, Nrf2 inhibitor ML385	Meng et al. (2025)
Water extract of Moschus (from Moschus berezovskii Flerov, Moschidae)	*In vitro*, Erastin-induced HT22 cells	30, 60 μg/mL	24 h	Regulated Keap1/Nrf2 pathway, ↑GPX4/SLC7A11/FTH1/FPN1, ↓TFRC, ↓MDA/ROS/LPO, ↑GSH	Model group, positive control Fer-1, Nrf2 inhibitor ML385	Song et al. (2024)

### 4.1 Effects of botanical drugs derived from TCM on AD through ferroptosis

#### 4.1.1 Alkaloids

Li et al. (2023b) found that BBR directly binds to Keap1 protein, blocking the Keap1-Nrf2 interaction, thereby activating Nrf2 nuclear translocation and the expression of its downstream target genes *ferroportin1 (FPN1)*, *solute carrier family seven member 11 (SLC7A11)*, *GPX4*, and *heme oxygenase-1 (H O -1)*, reducing iron overload and inhibiting lipid peroxidation. Meanwhile, BBR also enhances antioxidant defense via the AMPK/GSK3β/Nrf2 pathway, reverses mitochondrial cristae structural damage, and improves the pathological progression of AD.

#### 4.1.2 Glycosides

Salidroside (from *Rhodiola rosea* L., Crassulaceae) can inhibit neuronal ferroptosis in AD models by activating the Nrf2/HO-1 and Nrf2/GPX4 signaling axes. It mainly manifests by repairing mitochondrial cristae structure and membrane potential, reducing malondialdehyde (MDA), ROS levels, and Fe^2+^ accumulation, upregulating *GPX4 and SLC7A11* expression, and downregulating *transferrin receptor 1 (TFR1)* expression, thereby improving iron metabolism imbalance, inhibiting lipid peroxidation, and the subsequent neuroinflammation and oxidative stress. Additionally, salidroside can reduce CD8^+^ T cell infiltration and excessive activation of microglia, ultimately alleviating cognitive dysfunction (Yang et al., 2023). Forsythoside A (from *Forsythia suspensa* (Thunb.) Vahl, Oleaceae), a metabolite from Forsythia suspensa, has also been confirmed to inhibit ferroptosis by activating the Nrf2/GPX4 axis, thereby alleviating neuroinflammation (Wang C. et al., 2022). Both Curculigoside (from *Curculigo orchioides* Gaertn., Hypoxidaceae) and Senegenin (from *Polygala tenuifolia* Willd., Polygalaceae) can inhibit ferroptosis by regulating GPX4, thus improving AD (Zhang et al., 2022; Gong et al., 2024).

#### 4.1.3 Terpenoids

Research has shown that Paeoniflorin (from *Paeonia lactiflora* Pall., Paeoniaceae), a terpenoid metabolite from Paeonia lactiflora, can specifically bind to p53 and inhibit its expression, restoring the transcriptional activity of *SLC7A11* while upregulating the protein levels of superoxide dismutase (SOD) and GPX4. This mechanism significantly reduces ROS and iron ion levels in brain tissue, thereby blocking the p53-mediated oxidative stress response and inhibiting the ferroptosis process, ultimately improving neurodamage in AD mice (Zhai et al., 2023). Astragalus saponins (from *Astragalus membranaceus* (Fisch.) Bge., Fabaceae) inhibit the activity of NADPH oxidase 4 (NOX4) protein and reduce its stability, activate the Nrf2 signaling pathway and the expression of its downstream factors *H O -1, SLC7A11*, and *GPX4*, and coordinate the regulation of ferritin heavy chain 1/ferroportin1 (FTH1/FPN1)-mediated iron metabolism balance. Ultimately, they inhibits neuronal ferroptosis, alleviating Aβ/Tau pathology and cognitive dysfunction (Wang M. et al., 2025). Artemisinin (from *A. annua* L., Asteraceae), the well-known terpenoid metabolite from Artemisia annua, also targets the Nrf2 pathway, competitively binding KEAP1 to promote its nuclear translocation, upregulating SLC7A11 and GPX4, inhibiting lipid peroxidation and mitochondrial damage, and improving memory function in mice (Deng et al., 2025).

### 4.2 The effect of traditional Chinese medicine formulas on AD through ferroptosis

DSS activates AMPK phosphorylation, inhibits the transcriptional activity of *ACSL4* mediated by specific protein 1, reduces lipid peroxidation accumulation, upregulates the expression of key protective proteins in ferroptosis, and ultimately significantly improves cognitive dysfunction in model mice (Gong et al., 2025). Linggui Zhugan Decoction, from *Shanghan Lun*, a warming yang-based formula, can dose-dependently reduce oxidative stress markers MDA and Fe^2+^ levels, increase GSH content, and upregulate *GPX4 and SLC7A11* expression, thereby alleviating hippocampal neuronal morphological abnormalities and iron deposition, exerting neuroprotective effects (Fan et al., 2024).

Collectively, these findings reveal that traditional Chinese medicine modulates ferroptosis in AD through a multi-target network centered on the Nrf2 pathway. While certain alkaloids directly target Keap1 to activate Nrf2, many glycosides primarily upregulate Nrf2 downstream effectors, and various terpenoids engage parallel pathways including p53 inhibition and NOX4 suppression. This demonstrates that structurally diverse metabolites converge on common nodes while also engaging distinct auxiliary targets. Notably, traditional formulas such as DSS and Linggui Zhushugan Decoction exhibit combinatorial effects that simultaneously modulate multiple ferroptosis-related pathways, reflecting the multi synergy of botanical drugs. Together, these studies establish ferroptosis as a critical nexus linking traditional medicine mechanisms to modern neurobiology, and suggest that strategic combination of metabolites targeting complementary nodes may yield enhanced therapeutic efficacy in AD.

## 5 Autophagy and AD

**FIGURE 3:**
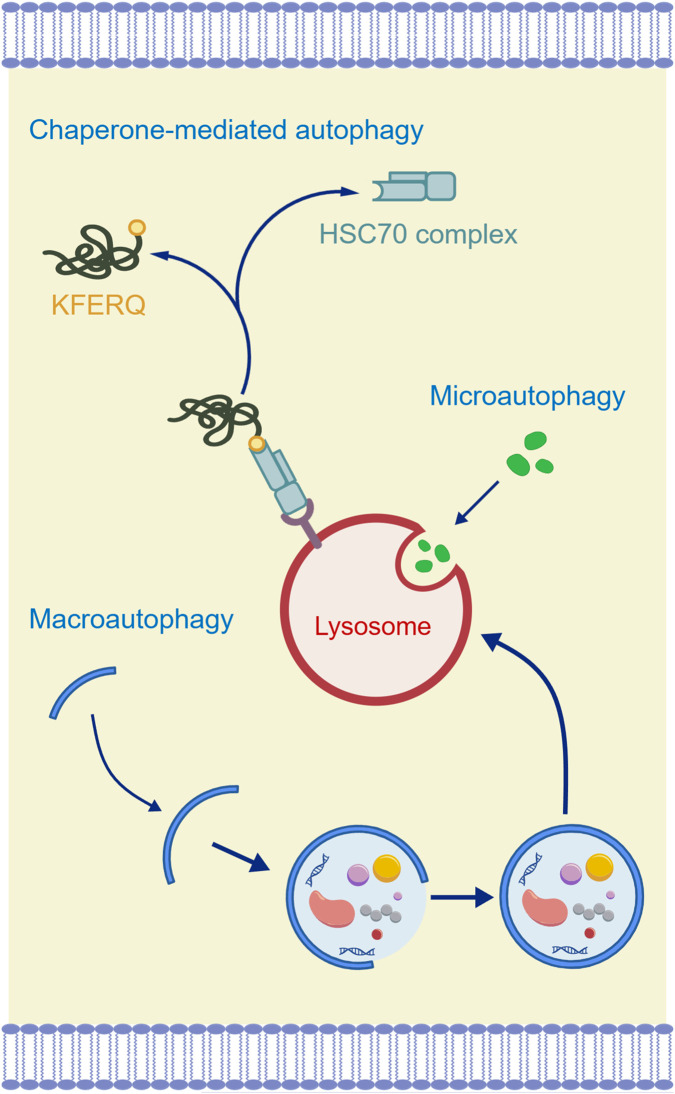
Mechanisms of autophagy. Created with BIOGDP.com (Jiang et al., 2025), licensed under CC BY NC.

Autophagy maintains cellular homeostasis through mTOR-dependent and -independent pathways (Figure 3). Under normal conditions, autophagy helps recycle intracellular substances to maintain cellular homeostasis, while excessive activation or functional defects of autophagy can lead to autophagy-related cell death (Ren et al., 2023). In Alzheimer’s disease (AD), autophagy exhibits bidirectional dysregulation—deficient autophagy leads to toxic protein accumulation, while hyperactivation exacerbates neuronal injury. Thus, precise modulation is critical. Traditional Chinese medicine (TCM) interventions should exert dose-dependent bidirectional effects: appropriately enhancing autophagy when it is insufficient, and inhibiting it when overactivated, rather than unidirectional promotion or suppression. The autophagy process relies on the lysosome to degrade damaged organelles, with mitophagy being particularly important for regulating mitochondrial quality in neurons. This process is mainly mediated by the PTEN-induced kinase 1/Parkin (PINK1/Parkin) pathway. When mitochondrial membrane potential is damaged, PINK1 accumulates on the outer mitochondrial membrane and recruits Parkin, leading to the ubiquitination of mitochondrial proteins, which promotes autophagosome formation and lysosomal degradation (Roca-Agujetas et al., 2021). In AD, Aβ can directly deposit on the mitochondrial membrane, inhibiting the activity of the electron transport chain and interfering with PINK1/Parkin pathway activation. It can also excessively activate autophagy but block autophagosome-lysosome fusion, leading to the accumulation of damaged mitochondria (Mary et al., 2023). At the same time, hyperphosphorylation of tau protein can block Parkin translocation to mitochondria, further inhibiting autophagic clearance. These mechanisms form a vicious cycle, exacerbating mitochondrial dysfunction and neurodegenerative lesions (Cummins et al., 2019). Therefore, targeting autophagy with TCM-derived metabolites and formulas represents a promising therapeutic strategy for AD (Table 3).

**TABLE 3:** Regulation of cell autophagy by botanical drugs.

Botanical drug/Metabolite	Model system	Dose/Concentration	Treatment duration	Mechanism and outcome	Controls used	References
Berberine	*In vivo*, 3×Tg-AD mice; *In vitro*, primary hippocampal neurons	*In vivo*: 100 mg/kg (drinking water); *In vitro*: 10 μM	*In vivo* 4 months; *In vitro* 24 h	Regulated Akt/GSK3β, ↑PP2A, ↓tau hyperphosphorylation, activated class III PI3K/Beclin-1 pathway, ↑autophagy, ↑cathepsin D, ↓total tau, ↑cognitive function	Model group, WT control, autophagy inhibitor 3-MA	Chen et al. (2020b)
Berberine + Curcumin	*In vivo*, APP/PS1 mice	BBR:100 mg/kg, CUR:200 mg/kg (oral)	3 months	Synergistic effects, ↓Aβ1-42, ↓inflammatory cytokines, ↓oxidative stress, ↓APP/BACE1, ↑AMPKα phosphorylation, ↑autophagy, ↑cognitive function	Model group, BBR alone, CUR alone	Lin et al. (2020)
Berbamine	*In vivo*: APP/PS1 mice; *In vitro*: LPS-stimulated BV2 cells	*In vivo*: 25,50 mg/kg (oral); *In vitro*: 4,8 μM	*In vivo* 3 weeks; *In vitro* 24 h	Targeted mTOR FRB domain, inhibited mTOR, ↑autophagy, promoted microglial M2 polarization, ↓inflammatory cytokines, ↑IL-4/IL-10, ↓Aβ deposition, ↑cognitive function	Model group, mTOR agonist MHY1485	Ge et al. (2025)
Panax quinquefolium saponins	*In vivo*: SAMP8 mice; *In vitro*: D-galactose-induced HT22 cells	*In vivo*: 100,200 mg/kg (oral); *In vitro*: 25,50 μg/mL	*In vivo* 10 weeks; *In vitro* 24 h	Activated AMPK/mTOR/ULK1/DRP1 and SIRT1/PGC-1α pathways, promoted PINK1/Parkin-mediated mitophagy, ↑LC3-II, ↓p62, ↓ROS, restored MMP, ↓p-Tau, ↑cognitive function	Model group (SAMR1), positive control donepezil, β-NMN	Zhao et al. (2025)
Senegenin	*In vitro*, Aβ1-42-induced HT22 cells	10,20,40,60 μM	24 h	Activated PINK1/Parkin pathway, ↑mitophagy, ↑PINK1/Parkin translocation to mitochondria, restored MMP, ↓ROS, ↓LDH, ↑cell viability	Model group, mitophagy inhibitor CsA, positive control CCCP	Tian et al. (2022)
Ciliatoside A	*In vitro*: LPS/Nig-stimulated BV2 cells; *In vivo*: 3xTg-AD mice, *C. elegans* AD models	*In vitro*: 2.5,5,10 μM; *In vivo*: 0.5,1,2 mg/kg (i.p.)	*In vitro*: 24 h; *In vivo*: Not specified	Activated AMPK/ULK1 and PINK1/Parkin pathways, ↑mitophagy, ↓NLRP3 inflammasome activation, ↓pyroptosis, ↓ROS, restored MMP, ↓Aβ deposition, ↓neuroinflammation, ↑cognitive function	Model group, positive controls MCC, Rap, CCCP, autophagy inhibitor Baf, AMPK inhibitor CC, Parkin inhibitor AC220, RNAi *C. elegans*	Guo et al. (2025)
1,8-Cineole	*In vivo*, *C. elegans* AD models (CL4176, CL2006, CL2355)	100–400 μM	36–48 h	Activated SKN-1/Nrf-2 pathway, ↑autophagy (↑LGG-1 puncta), ↓ROS, ↓Aβ aggregation, delayed Aβ-induced paralysis, improved chemotaxis	Model group, positive control quercetin	Tan et al. (2024)
Andrographolide derivative ADA	*In vivo*: Apoe4 mice; *In vitro*: Apoe4-transfected BV2, HT22 cells	*In vivo*: 1,2.5,5 mg/kg (i.p.); *In vitro*: 2 μM	*In vivo* 21 d; *In vitro* 24 h	Activated SIRT3-FOXO3a pathway, ↑PINK1/Parkin and BNIP3-mediated mitophagy, ↓LC3II/p62/TOM20 accumulation, ↓NLRP3 inflammasome activation, ↓inflammatory cytokines, ↓Aβ deposition, ↑cognitive function	Model group (Apoe3), SIRT3 inhibitor 3-TYP	Zhou et al. (2024)
Liuwei Dihuang Pill	*In vivo*, APP/PS1 mice	1.365 g/kg (oral)	60 d	Activated PI3K/Akt pathway, ↑autophagy, ↓Aβ deposition, ↓neuroinflammation, ↑cognitive function	Model group, positive control donepezil	Yuan et al. (2023)
Danggui Shaoyao San	*In vivo*, Aβ1-42-induced AD mouse model (C57BL/6J mice, both sexes, 8-week-old)	DSS: 6.4 g/kg/day; SG: 4.8 g/kg/day; XG: 4.0 g/kg/day (oral gavage)	28 days	Sham group (ICV saline); Model group (ICV Aβ1-42 + saline); Positive control: Donepezil (3 mg/kg/day)	Sham group (ICV saline); Model group (ICV Aβ1-42 + saline); Positive control: Donepezil (3 mg/kg/day)	Cheng et al. (2024b)
Yuan-Zhi Decoction	*In vivo*, BCCAO rat model (CCH); *In vitro*, primary rat hippocampal neurons (hypoxia-hypoglycemia)	*In vivo*: 5, 10, 20 g/kg/d; *In vitro*: 10% YZD serum, 8 μM TEN, 36 μM β-asarone	*In vivo*: 4 weeks; *In vitro*: 24 h	↑ Cognition (MWM, passive avoidance); ↓ Aβ deposition; ↓ autophagy (ATG5, ATG12, Beclin-1, LC3-II); ↓ BACE1, PS-1; ↑ neuronal survival	*In vivo*: Sham; Model; Donepezil. *In vitro*: Control; Model; Rapamycin; 3-MA.	Liu et al. (2020)
Triptolide (Tripterygium wilfordii Hook.f., Celastraceae)	*In vitro*, differentiated PC12 cells treated with Aβ1-42 (10 μmol/L)	0.1 nM, 1 nM, 10 nM	24 h	↑Cell viability; ↓apoptosis; ↑synaptic proteins; ↓Aβ metabolism; ↓autophagosome accumulation; ↑autophagic degradation; activated Akt/mTOR/p70S6K pathway	Untreated control; Aβ1-42 model (10 μmol/L); Pathway inhibitor controls: Akt-I-1/2, rapamycin, mTOR siRNA	Xu et al. (2022)
Aloe-emodin (Rheum palmatum L./Aloe vera (L.) Burm.f.)	*In vivo*, APP/PS1 mice (6-month-old, male); *In vitro*, HT22 cells (Aβ25-35, 30 μM)	*In vivo*: 25, 50, 100 mg/kg (i.g.); *In vitro*: 2, 4, 6 μM	*In vivo*: 28 d; *In vitro*: 24 h	↑ Cognitive function; ↓ hippocampal neuronal damage; activated mitophagy; improved mitochondrial function; activated AMPK/PGC-1α/SIRT3 pathway. Effects reversed by SIRT3 siRNA.	*In vivo*: WT (C57BL/6J), Model (APP/PS1), Positive: Donepezil (2.1 mg/kg). *In vitro*: Control, Aβ model, Positive: Rapamycin (200 nM). Mechanism: SIRT3 siRNA	Wang et al. (2025b)
Aloe-emodin (Rheum palmatum L./Aloe vera (L.) Burm.f.)	*In vitro*, differentiated PC12 cells (rat) treated with glutamate (20 mM)	0.05, 0.1 mg/mL	24 h pretreatment +24 h co-treatment	↑ Cell viability; ↓ LDH release; ↓ apoptosis; ↓ mitochondrial damage; ↓ autophagic vacuoles/autophagosomes; regulated autophagy	Basic: Untreated control; Glu model (20 mM). Positive: 3-MA (5 mM, autophagy inhibitor)	Xie et al. (2023)
Ethyl acetate extract of Curcuma wenyujin Y.H.Chen & C.Ling (Zingiberaceae	*In vivo*, Aβ1-42 ICV mice (male Kunming); *In vitro*, SH-SY5Y cells (Aβ1-42)	*In vivo*: 18.2 mg/kg (EAC, i.g.); *In vitro*: 10, 20, 40 μg/mL	*In vivo*: 21 d; *In vitro*: 24 h	↑Cognitive function; ↓neuronal damage and apoptosis; ↓Aβ deposition; ↓pyroptosis; ↑ mitophagy; activated PINK1/Parkin pathway. Effects reversed by PINK1 siRNA.	*In vivo*: Control (saline ICV), Model (Aβ ICV), Positive: Huperzine A, Other fractions: TT, PE, NB, WA. *In vitro*: Control, Aβ model, Positive: Rapamycin. Mechanism: PINK1 siRNA	Qi et al. (2025)

### 5.1 Effects of botanical drugs derived from TCM on AD through autophagy

#### 5.1.1 Alkaloids

Studies have shown that BBR can activate the PI3K/myosin-like BCL2 interacting protein (Beclin-1)/Bcl-2 autophagy pathway, promoting autophagosome formation and autophagic flux activation, thereby promoting the degradation and clearance of abnormal tau protein through the autophagy-lysosome pathway (Chen Y. et al., 2020). It is worth noting that combining BBR with curcumin (from *Curcuma longa* L., Zingiberaceae) produces a synergistic effect, reflecting the scientific essence of TCM compatibility (Lin et al., 2020). Berbamine hydrochloride, an alkaloid metabolite derived from Phellodendri Chinensis Cortex (*Phellodendron amurense* Rupr., Rutaceae), targets the FKBP12-rapamycin-binding domain of mTOR to inhibit the mTOR complex one signaling pathway. This effect not only activates the autophagy process but also promotes microglial polarization to the anti-inflammatory M2 type, thereby enhancing Aβ clearance and alleviating neuroinflammation (Ge et al., 2025).

#### 5.1.2 Glycosides

Panax quinquefolium saponins, a class of glycoside metabolites derived from American Ginseng Radix (*Panax quinquefolius* L., Araliaceae), as core metabolites from traditional tonic botanical drugs used in TCM, have been confirmed to promote PINK1/Parkin-mediated mitophagy by activating the AMPK/mTOR/Unc-51 like autophagy activating kinase 1 (ULK1)/dynamin-related protein 1 (DRP1) and SIRT1/PGC-1α signaling pathways. This improves mitochondrial dysfunction, reduces ROS accumulation in neurons and Tau hyperphosphorylation, alleviates hippocampal pathology, and improves memory function in animal models (Zhao et al., 2025). Polygala saponins (*P. tenuifolia* Willd., Polygalaceae) can also promote PINK1/Parkin-mediated mitophagy, improving mitochondrial membrane potential and oxidative stress, thereby inhibiting neuronal apoptosis (Tian et al., 2022). Ciliatoside A, a glycoside metabolite derived from *Peristrophe japonica* (Thunb.) Bremek. (Acanthaceae), not only activates the PINK1/Parkin pathway but also upregulates AMPK/ULK1 signaling, further enhancing autophagic flux and inhibiting NLRP3 inflammasome activation and microglial pyroptosis (Guo et al., 2025).

#### 5.1.3 Terpenoids

1,8-Cineole, a monoterpenoid metabolite prominently found in Amomi Fructus (the dried fruit of Amomum villosum Lour., Zingiberaceae) as well as in other aromatic plants worldwide, can activate the SKN-1/Nrf-2 antioxidant pathway and induce autophagy, reducing Aβ deposition and ROS levels, thereby delaying the paralysis phenotype induced by Aβ. Of note, as a volatile constituent, 1,8-cineole faces inherent challenges in stability and formulation. To address this, further research employed β-cyclodextrin encapsulation technology to prepare 1,8-cineole complexes, which significantly improved its bioavailability and anti-AD activity. This strategy not only validates the therapeutic potential of 1,8-cineole but also provides a valuable formulation reference for improving the delivery of volatile metabolites in TCM for AD treatment (Tan et al., 2024). Andrographolide derivative ADA, synthesized from andrographolide derived from Andrographidis Herba (*Andrographis paniculata* (Burm.f.) Nees, Acanthaceae), can activate the SIRT3-FOXO3a pathway, effectively repairing damaged mitochondrial autophagy functions, promoting PINK1/Parkin-dependent mitochondrial clearance, and enhancing the BNIP3 receptor-mediated mitophagy pathway. This suppresses the overactivation of inflammasomes and neuroinflammatory responses, ultimately reducing Aβ deposition, neuronal damage, and improving cognitive dysfunction (Zhou et al., 2024).

### 5.2 The effect of traditional Chinese medicine formulas on AD via autophagy

Liuwei Dihuang Pills, based on the TCM theory of “nourishing the kidney and replenishing essence”, exert neuroprotective effects through the PI3K/Akt pathway. This formula reduces Aβ deposition in the hippocampus, inhibits microglial and astrocyte activation, lowers pro-inflammatory factors such as interleukin-1β (IL-1β), and promotes neuronal autophagy by upregulating Beclin-1 expression and the LC3-II/LC3-I ratio, thereby alleviating neuroinflammation and neuronal damage (Yuan et al., 2023). DSS can protect hippocampal neurons by activating PINK1-Parkin-mediated mitophagy and inhibiting mitochondrial apoptosis (Song et al., 2021). Further research shows that DSS and its subformulas (SG, XG) can activate the AMPK/mTOR pathway, downregulate APP and p-Tau proteins, and upregulate post-synaptic density protein-95 to repair synaptic damage and inhibit neuroinflammation. Among them, DSS as a whole is more effective than its subformulas, reflecting the overall advantage of the combined formula (Cheng X. et al., 2024).Chronic cerebral hypoperfusion (CCH) is a major risk factor and common comorbidity in AD. Under CCH conditions, autophagy is aberrantly overactivated, exacerbating Aβ deposition and neuronal injury, thereby accelerating AD progression. Research reported that in a rat model of CCH induced by bilateral common carotid artery occlusion, treatment with Yuanzhi decoction (composed of Polygalae Radix and Acori Tatarinowii Rhizoma at a 1:1 ratio) at doses of 5, 10, and 20 g/kg/d for 4 weeks dose-dependently improved cognitive function and significantly reduced the expression of autophagy-related proteins ATG5 and ATG12 in the hippocampus, thereby inhibiting pathological overactivation of autophagy. In an *in vitro* model of oxygen-glucose deprivation (OGD)-induced injury in primary hippocampal neurons, treatment with 10% Yuanzhi-containing serum, as well as its active metabolites tenuigenin (8 μM) and β-asarone (36 μM), decreased the expression of Beclin-1 and LC3-II and reduced Aβ1-40 and Aβ1-42 deposition. These findings suggest that inhibiting excessive autophagy under AD-related pathological conditions may confer neuroprotective benefits, offering a novel therapeutic strategy for AD (Liu et al., 2020).

Collectively, the aforementioned studies suggest that TCM can intervene in the pathological progression of AD by modulating autophagy. Autophagy exhibits a dose-dependent biphasic effect: moderate activation facilitates the clearance of toxic protein aggregates, whereas sustained or excessive activation may trigger autophagic cell death. Therefore, future research should integrate dynamic monitoring of autophagy flux to systematically evaluate the dose-effect-toxicity relationships of active metabolites and formulas derived from TCM, thereby identifying optimal therapeutic concentrations and intervention windows. Elucidating this critical scientific question is essential for advancing TCM-based autophagy modulation strategies from fundamental research to clinical translation, and may offer novel insights for precision therapy in AD.

## 6 Pyroptosis and AD

**FIGURE 4:**
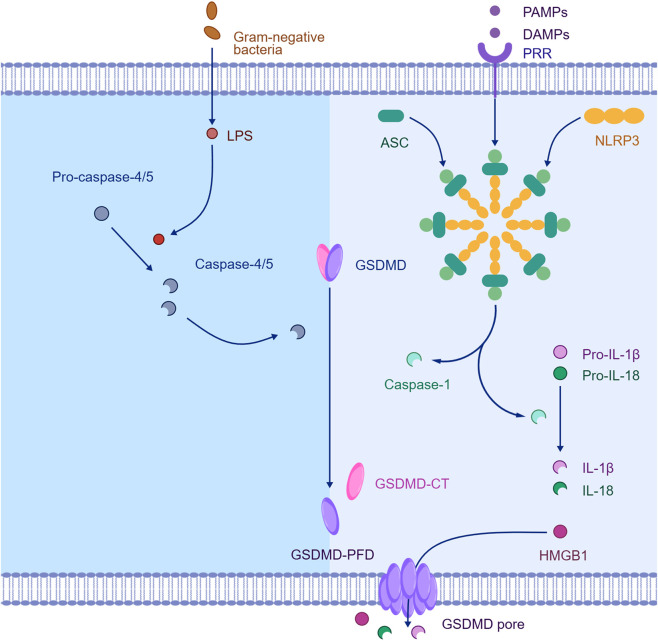
Mechanisms of pyroptosis. Created with BIOGDP.com (Jiang et al., 2025), licensed under CC BY NC.

Cell pyroptosis is an inflammatory programmed cell death (PCD) executed by the activation of inflammasomes and gasdermin family proteins, involving both classical and non-classical pathways (Liu X. et al., 2024) (Figure 4). The classical pathway is activated by NLRP1, NLRP3, or AIM2 inflammasomes, which activate caspase-1. Caspase-1 then cleaves gasdermin D (GSDMD), forming membrane pores that cause cell rupture and promote the maturation and release of pro-IL-1β and pro-IL-18. The non-classical pathway is activated by lipopolysaccharide (LPS), which directly binds and activates caspase-4/5/11, leading to GSDMD cleavage and triggering pyroptosis (Rao et al., 2022). In AD, Aβ can induce neuronal pyroptosis by activating the NLRP3-caspase-1-GSDMD axis, promoting neuroinflammation and neuronal damage, thus accelerating AD progression (Bai et al., 2021). Meanwhile, NLRP3 can exacerbate neuroinflammation and promote Aβ deposition by activating caspase-1 and releasing inflammatory factors such as IL-1β (Liang et al., 2020). At the same time, tau hyperphosphorylation can activate caspase-1 and increase inflammatory factor levels, further promoting pyroptosis. Inhibiting caspase-1 or reducing tau phosphorylation can alleviate pyroptosis and neuronal damage, indicating a vicious cycle between the two that jointly drives neurodegeneration in AD (Li Y. et al., 2020).

### 6.1 Effects of botanical drugs derived from TCM on AD via pyroptosis

Dendrobium nobile alkaloids (*Dendrobium nobile* Lindl., Orchidaceae), the main active metabolites of Dendrobium, can inhibit the activation of *NLRP3* inflammasomes, reduce caspase-1 activity, and thus decrease GSDMD cleavage and the formation of the pyroptotic executor protein GSDMD-N, alleviating LPS-induced hippocampal neuronal pyroptosis and cognitive dysfunction. It did not show the same protective effect in *NLRP3* knockout mice, further indicating that its neuroprotective effect relies on the inhibition of the NLRP3/GSDMD signaling pathway (Li et al., 2022) (Table 4). Salidroside (*R. rosea* L., Crassulaceae) can also directly inhibit the core pyroptotic signaling pathway of NLRP3/Caspase-1, but it can additionally modulate NLRP3 inflammasome activation indirectly through its upstream TLR4/NF-κB/MyD88 signaling axis, thereby significantly reducing Aβ deposition, tau hyperphosphorylation, and neuronal damage, lowering the release of inflammatory factors such as IL-1β and IL-18, and improving cognitive dysfunction (Cai et al., 2022). Schisandrin (*Schisandra chinensis* (Turcz.) Baill., Schisandraceae), significantly inhibits Aβ-induced NLRP1 inflammasome activation, subsequently inhibiting caspase-1 cleavage and the maturation and release of downstream inflammatory factors IL-1β and IL-18, effectively blocking the neuronal pyroptosis pathway. At the same time, it alleviates the neuroinflammatory environment and indirectly inhibits neuronal apoptosis, ultimately improving cognitive function in AD model animals (Li et al., 2021).

**TABLE 4:** Regulation of cell pyroptosis by botanical drugs.

Botanical drug/Metabolite	Model system	Dose/Concentration	Treatment duration	Mechanism and outcome	Controls used	References
Dendrobium nobile alkaloids	*In vivo*, LPS hippocampal injection mouse model (WT and NLRP3 KO)	20, 40 mg/kg (i.g.)	14 days (LPS on day 7)	↑Cognition; protected neurons); inhibited pyroptosis (↓GSDMD-N, caspase-1) and NLRP3 inflammasome	WT control; WT LPS; NLRP3 KO control; NLRP3 KO LPS	Li et al. (2022)
Salidroside	*In vivo*, 2 mouse models: Aβ1-42 hippocampal injection; D-gal/AlCl_3_ induced. *In vitro*, PC12 cells (D-gal or Nigericin)	*In vivo*: 20, 40, 80 mg/kg (i.g.); *In vitro*: 2, 10, 50 μM	*In vivo*: 14 d (Aβ model) or 10 weeks (D-gal/AlCl_3_); *In vitro*: 48 h	↑Cognition; ↓Aβ and p-tau; inhibited NLRP3 pyroptosis (↓IL-1β, IL-18, GSDMD-N, NLRP3, ASC, caspase-1) via TLR4/NF-κB pathway	*In vivo*: Control; Model; Donepezil. *In vitro*: Control; D-gal; Nigericin	Cai et al. (2022)
Schisandrin	*In vivo*, APP/PS1 mice (12-month-old); *In vitro*, SH-SY5Y cells (Aβ1-42, 10 μmol/L)	*In vivo*: 2 mg/kg/d (i.g.); *In vitro*: 10 μmol/L	*In vivo*: 2 weeks; *In vitro*: 24 h	↑Cognition; ↓Aβ and neuronal apoptosis (↓caspase-3, Bax; ↑Bcl-2); inhibited NLRP1 pyroptosis (↓NLRP1, ASC, caspase-1, IL-1β, IL-18)	*In vivo*: WT; APP/PS1. *In vitro*: Control; Aβ	Li et al. (2021)
Jiedu Yizhi Formula	*In vivo*, Aβ25-35 bilateral hippocampal injection rat model	3.6, 7.2, 14.4 g/kg/d (crude drug, i.g.)	8 weeks	↑Cognition; ↓Aβ deposition; protected hippocampal neurons; inhibited pyroptosis via NLRP3/Caspase-1/GSDMD and LPS/Caspase-11/GSDMD pathways; ↓IL-1β, IL-18 in multiple tissues	Sham (saline ICV); Model (Aβ ICV); Positive: Donepezil (0.9 mg/kg)	Wang et al. (2022c)
Jiedu Yizhi Formula	*In vivo*, Aβ25-35 bilateral hippocampal injection rat model	3.6, 7.2, 14.4 g/kg/d (i.g.)	8 weeks	Modulated gut microbiota (↑Bacteroidota, *Lactobacillus*; ↓Firmicutes, *Helicobacter*); ↓hippocampal pyroptosis (↓Caspase-1, Caspase-11)	Sham (saline ICV); Model (Aβ ICV); Positive: Donepezil (0.9 mg/kg)	Wang et al. (2022b)
Jiedu Yizhi Formula	*In vivo*, APP/PS1 mice	10.536, 20.268 g/kg/d (i.g.)	8 weeks	↑Cognition; ↓p-tau; modulated gut microbiota (↑Lachnospiraceae; ↓Alistipes); inhibited TLR4/NF-κB pathway	WT (C57BL/6); Model (APP/PS1); Positive: Donepezil (0.45 mg/kg)	Zhang et al. (2023a)

### 6.2 The effect of traditional Chinese medicine formulas on AD via pyroptosis

Detoxifying and Intelligence-enhancing Prescription, proposed by renowned TCM master Ren Jixue, improves cognitive function primarily through the suppression of neuroinflammation and pyroptosis. Studies have confirmed that this formula acts directly on hippocampal tissue, inhibiting both the NLRP3/caspase-1/GSDMD and LPS/caspase-11/GSDMD pyroptosis pathways, reducing Aβ deposition in hippocampal tissue, and lowering the release of inflammatory factors IL-1β and IL-18, thereby alleviating neuroinflammation and improving cognitive function (Wang et al., 2022c). Further research has revealed its systemic regulatory effect. The Detoxifying and Intelligence-enhancing Prescription can regulate the gut microbiota composition in AD model animals, increasing beneficial bacteria, reducing pro-inflammatory bacteria, and indirectly inhibiting the activation of Caspase-1 and Caspase-11 in the hippocampus, thereby enhancing its anti-pyroptosis and anti-inflammatory effects (Wang et al., 2022b). Additionally, the formula, through its reshaped gut microbiota, can also inhibit the activation of the hippocampal TLR4/NF-κB signaling pathway, reduce the expression of downstream inflammatory factors, and alleviate the abnormal deposition of phosphorylated tau proteins, thereby exerting neuroprotective and cognitive-improving effects through multiple synergistic mechanisms (Zhang P. et al., 2023).

In summary, traditional Chinese medicine intervenes in AD-related pyroptosis through both canonical and non-canonical pathways (Table 5). Certain alkaloids and extracts inhibit NLRP3 activation, while other metabolites target the NLRP1 inflammasome. Traditional formulas exhibit multi-level regulation: they not only directly suppress pyroptotic pathways in the hippocampus but also indirectly modulate pyroptosis via gut microbiota remodeling and the TLR4/NF-κB axis, reflecting the holistic regulatory advantage based on the gut-brain axis.

**TABLE 5:** Composition and preparation of representative traditional Chinese medicine formulas.

Formula name	Composition	Preparation method
Shenghui Decoction	Rehmanniae Radix Praeparata (Orobanchaceae) Corni Fructus (Cornaceae) Polygalae Radix (Polygalaceae) Ziziphi Spinosae Semen (Rhamnaceae) Platycladi Semen (Cupressaceae) Poria cum Radix Pini (Polyporaceae) Ginseng Radix et Rhizoma (Araliaceae) Acori Tatarinowii Rhizoma (Acoraceae) Sinapis Semen (Brassicaceae)	The medicinal materials were crushed into small pieces, decocted with 10 times the amount of water (1:10 w/v) at 100 °C for 1 h. This process was repeated 3 times. The filtrates were combined, concentrated, freeze-dried into a powder, and stored in a desiccator at −20 °C
Danggui Shaoyao San	Angelica sinensis (Oliv.) Diels (Apiaceae) Paeonia lactiflora Pall (Paeoniaceae) Atractylodes macrocephala Koidz (Asteraceae) Poria cocos (Schw.) Wolf (Polyporaceae) Alisma orientale (Sam.) Juzep (Alismataceae) Ligusticum chuanxiong Hort (Apiaceae) Ratio: 3:16:4:4:8:8 (Angelica:Paeonia:Atractylodes:Poria:Alisma:Ligusticum)	Prepared by water decoction, followed by concentration and lyophilization to obtain a standardized extract
Linggui Zhugan Decoction	Cinnamomi Ramulus (Lauraceae) Poria cocos (Schw.) Wolf (Polyporaceae) Atractylodes macrocephala Koidz. (Asteraceae) Glycyrrhizae Radix et Rhizoma (Fabaceae) Ratio: 3:4:3:2 (Ramulus Cinnamomi:Poria cocos:Atractylodes macrocephala:Glycyrrhizae Radix et Rhizoma)	Prepared by water decoction
Liuwei Dihuang Wan	Rehmannia glutinosa Libosch. (Scrophulariaceae) 8 Cornus officinalis Sieb. (Cornaceae) 4 Dioscorea opposita Thunb. (Dioscoreaceae) 4 Alisma orientale (G. Samuelsson) Juz (Alismataceae) 3 Poria cocos (Schw.) Wolf (Polyporaceae) 3 Paeonia suffruticosa Andrews (Paeoniaceae) 3 Ratio: 8:4:4:3:3:3 (total dry weight: 1 kg)	The mixed botanical drugs were decocted in 4 L distilled water for 30 min twice. The combined extracts were concentrated to a concentration of 1 g crude drug/mL for use
Jiedu Yizhi Formula	Coptis chinensis Franch. (Ranunculaceae) 1 part Rhei Radix et Rhizoma (wine-treated) (Polygonaceae) 1 part Ligusticum chuanxiong Hort. (Apiaceae) 1 part Pheretima (Megascolecidae) 1 part Testudinis Carapacis et Plastri Colla (Geoemydidae) 1 part Cornus officinalis Sieb. et Zucc. (Cornaceae) 1 part Alpinia oxyphylla Miq. (Zingiberaceae) 2 parts Ratio: 1:1:1:1:1:1:2	The botanical drugs were mixed according to the specified ratio, soaked in 5 volumes of distilled water for 1 h, boiled for 1 h, and then boiled a second time. The extraction solutions from both boils were combined and concentrated to a concentration of 1.0 g crude drug/mL. The final extract was placed in sterile containers, sealed, and stored at −20 °C

## 7 Translational hurdles in TCM-Based AD research

Despite the promising multi-target regulatory effects of TCM-derived metabolites on PCD pathways summarized above, several critical limitations must be acknowledged before these findings can be translated into clinical applications for AD.

First, a significant number of the bioactive metabolites discussed—including flavonoids, alkaloids, and certain polyphenols—are recognized as pan-assay interference metabolites (PAINS). These metabolites exhibit redox activity, intrinsic fluorescence, and a propensity for aggregation—properties that can interfere with biochemical and cellular assays, potentially yielding false-positive results (Baell, 2016). Consequently, some of the reported pleiotropic effects may reflect assay artifacts rather than genuine pharmacological actions. Confirmation of target engagement using orthogonal techniques—such as surface plasmon resonance, cellular thermal shift assays, or genetic loss-of-function models—is urgently needed to validate mechanistic claims. Second, the preclinical evidence base relies heavily on models with limited translational validity. *In vitro* systems lack the complexity of neural circuits, glial interactions, and the blood–brain barrier, while *in vivo* transgenic models are predominantly based on rare familial AD mutations and fail to capture sporadic, late-onset AD with its multifactorial etiology. These models do not completely replicate the full spectrum of human AD pathology, particularly its multifactorial etiology, progressive course, and age-related neurodegeneration (Alexandra Lopes and Guil-Guerrero, 2025). Future studies should employ multi-model validation, incorporate pharmacokinetic assessments, and leverage emerging human-relevant models to bridge the translational gap. Third, While our review highlights the promising multi-target effects of various TCM-derived metabolites on PCD pathways, it is crucial to acknowledge that the translational potential of these compounds, including those discussed above, is often constrained by pharmacokinetic challenges. Issues such as poor oral bioavailability, metabolic instability, and most importantly, the ability to cross the blood-brain barrier (BBB), remain universal hurdles (Kato et al., 2025). The example of 1,8-cineole and its cyclodextrin inclusion complex serves as a paradigm for this challenge; despite its potent *in vivo* activity in *C. elegans*, its volatile nature necessitates advanced formulation strategies to ensure stability and efficacy. For other metabolites like glycosides and alkaloids, similar obstacles exist. Therefore, future research must move beyond merely identifying bioactive compounds to systematically evaluating and improving their druggability. This includes the development of brain-targeted delivery systems, such as nanoparticles, liposomes, or engineered exosomes, to enhance BBB penetration and ensure targeted delivery to affected neurons. Only by integrating formulation science with pharmacological evaluation can the true therapeutic potential of these TCM-inspired leads be realized in a clinical setting.

## 8 Conclusion and future perspectives

In summary, the PCD mechanism plays a key role in the progression of AD, and metabolites derived from TCM botanical drugs, with their multi-target and holistic regulation advantages, can intervene in these processes to delay AD development. It is noteworthy that the course of AD shows significant stage-dependent heterogeneity, which means that intervention strategies inspired by TCM principles should also be differentiated: in the early stage of cognitive impairment, the focus may be on regulating autophagy and inhibiting inflammation-related pyroptosis to protect neuronal function; while in the later stage of dementia, greater emphasis should be placed on inhibiting apoptosis and ferroptosis to alleviate established neuronal damage. Achieving such precise regulation based on disease stages is an important development direction for TCM-inspired approaches in the prevention and treatment of AD, but it still faces practical challenges such as unclear mechanisms, uncertain active metabolites, and insufficient clinical translation.

Future research should integrate methods such as systems pharmacology, multi-omics, and artificial intelligence to further elucidate the mechanisms of TCM formulas in the synergistic regulation of PCD networks. At the same time, efforts should be made to develop brain-targeted delivery strategies, drawing on successful experiences of engineered exosomes carrying siRNA across the blood-brain barrier to improve the brain delivery efficiency of bioactive metabolites derived from botanical drugs. Moreover, a multi-level research system from basic research to clinical studies should be established to form a complete evidence chain, ultimately promoting the development of theoretical frameworks and practical guidelines for the prevention and treatment of AD using TCM-inspired approaches.

This review highlights how investigating natural products, identified through the lens of TCM’s clinical experience and theoretical framework, can reveal novel multi-target mechanisms for AD. While the metabolites themselves are part of a shared global natural heritage, the TCM-inspired approach—which emphasizes network regulation and the synergistic action of multiple metabolites—provides a valuable paradigm for developing next-generation therapeutics that can simultaneously modulate the complex PCD networks implicated in AD.
